# Quaternary Ammonium Compounds and Sodium Hypochlorite for the Control of Bacterial Contamination on Hatching Eggshells: A Review

**DOI:** 10.3390/ani16132104

**Published:** 2026-07-07

**Authors:** Gabriel da Silva Oliveira, Luana Maria de Jesus, Concepta McManus, Vinícius Machado dos Santos

**Affiliations:** 1 Faculty of Veterinary Medicine and Animal Science, University of São Paulo, São Paulo 05508-270, Brazil; 2 Faculty of Agronomy and Veterinary Medicine, University of Brasília, Brasília 70910-900, Brazil; 3 Laboratory of Poultry Science, Federal Institute of Brasília—Campus Planaltina, Brasília 73380-900, Brazil; 4 Center for Nuclear Energy in Agriculture (CENA), University of São Paulo, São Paulo 13416-000, Brazil

**Keywords:** bacterial control, egg microbiology, hatching eggs, poultry sanitizers, synthetic products, table eggs

## Abstract

Sanitization constitutes a pillar of modern bacterial control in the management of hatching eggs. When improperly performed, eggshell contamination may increase, thereby increasing embryo susceptibility to bacterial pathogens and negatively affecting their viability and development. Sanitizing agents based on quaternary ammonium compounds (QACs) and sodium hypochlorite (NaClO) have been considered potential treatments to mitigate bacterial problems in poultry production. Therefore, this review aims to analyze the antibacterial potential of QACs and NaClO as sanitizers for bacterial control on hatching eggshells. In summary, both evaluated compounds proved effective in reducing bacterial contamination on eggshell surfaces, as evidenced by the significant antibacterial activity observed. However, their use should be rational, respecting the specific characteristics and recommendations of each compound, and guided by manufacturers’ guidelines and scientific evidence.

## Simple Summary

Due to the high risks of production losses associated with bacterial contamination in poultry production systems, the strategic management of egg sanitization is essential for improving microbiological safety and supporting poultry production. Various antibacterial compounds are currently available for use in poultry management. This review aims to analyze the antibacterial potential of QACs and NaClO as sanitizers for bacterial control on hatching eggshells.

## 1. Introduction

The horizontal transmission of pathogenic bacteria from poultry surfaces, such as nests or bedding, which may harbor microorganisms including *Staphylococcus* spp., total coliforms, thermotolerant coliforms, mesophilic and psychrotrophic bacteria, *Clostridium* spp., and *Salmonella* spp., to newly laid hatching eggs contributes to infections associated with the production environment [1]. Omphalitis, for example, characterized by infection of the yolk sac, mainly by *Escherichia coli*, may be associated with bacterial contamination of the eggshell [2,3]. Therefore, sanitization of hatching eggs is a recommended step after egg collection.

Commercial chemical agents containing quaternary ammonium compounds (QACs) have been tested in sanitization protocols for hatching eggs. Chan et al. [4] reported that sanitization with QACs at concentrations of 100 and 200 ppm reduced aerobic mesophilic bacteria on the eggshell by approximately 4.0 log_10_ CFU/cm^2^, regardless of exposure time (5 or 15 min). Similarly, Laban et al. [5] reported that a sanitizer containing glutaraldehyde and QACs (0.5%) significantly reduced total bacterial and coliform counts on the eggshell surface of hatching eggs from nest and floor sources, regardless of breeder flock age (37, 47, and 57 weeks). Therefore, it appears that formulations containing QACs, when applied under different protocols, have demonstrated good efficacy in reducing bacterial loads on eggshell surfaces.

Sodium hypochlorite (NaClO) is another commercially available sanitizing agent that has been investigated for sanitizing hatching eggs. de Souza et al. [6] observed that the mean count of mesophilic aerobic bacteria on eggshells treated with NaClO after 24 h of refrigerated storage was 1.3 log_10_ CFU/mL, corresponding to an approximate 60.12% reduction compared with untreated eggs, which presented 3.26 log_10_ CFU/mL. Consistent with these findings, Zang et al. [7] demonstrated that, at an available chlorine concentration of 26 mg/L, the decontamination of shelled eggs through the application of NaClO for 3 min resulted in the complete inactivation of *Salmonella* Enteritidis and *Escherichia coli* on the eggshell surface. Like QACs, NaClO also appears to be a viable approach for controlling bacterial contamination on the eggshell surface.

Given the favorable cost–benefit profile of these sanitizing agents and their widespread use as antibacterial compounds in the food industry and animal production systems, we reviewed the potential of QACs and NaClO as sanitizers for bacterial control on hatching eggshells, based on evidence reported in studies published between 2016 and 2025, using data retrieved from Google Scholar.

## 2. Bacterial Contamination of Eggshells and Embryonic Infection Risk

**Figure 1:**
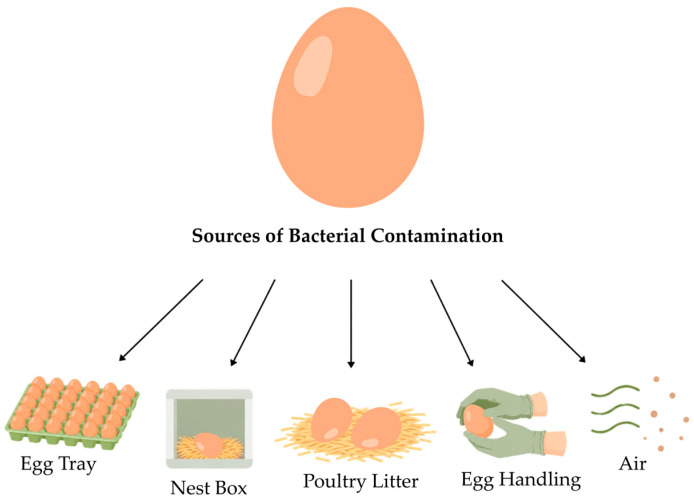
Potential sources of bacterial contamination of eggshell surfaces.

Bacterial contamination of eggs occurs predominantly after oviposition, and nests and litter contribute substantially to this bacterial load, as they are environments naturally colonized by diverse microorganisms [1]. Once the eggshell is contaminated, bacteria may penetrate the egg’s interior, thereby characterizing horizontal transmission. In this context, one of the main risk factors for pathogenic bacterial contamination of hatching eggs is the duration of contact with contaminated surfaces, mainly those with high accumulations of organic matter and debris. This explains why eggs laid on poultry litter (considered visibly dirty eggs), with a greater potential to serve as reservoirs of pathogenic bacteria, exhibit higher levels of contamination and a greater potential for the dissemination of these microorganisms [1,8]. However, other environmental surfaces may also serve as sources of eggshell bacterial contamination (Figure 1).

**Figure 2:**
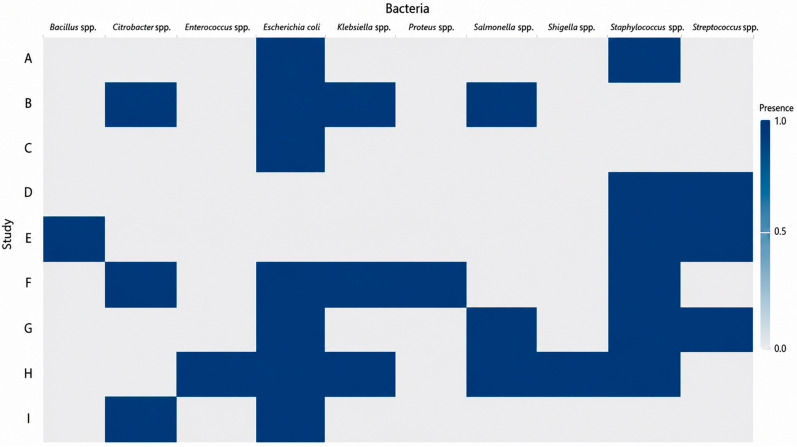
Occurrence of bacterial taxa on poultry eggshells. Blue cells indicate that the bacterial taxon was reported in the respective study, whereas white cells indicate that it was not reported. Source: Study A, Cortés et al. [12]; Study B, Fardows and Shamsuzzaman [13]; Study C, Mezhoud et al. [14]; Study D, Batkowska et al. [15]; Study E, Batkowska et al. [16]; Study F, Addo et al. [17]; Study G, Al-Shammari et al. [18]; Study H, Hassan et al. [19]; Study I, Mehmood et al. [20].

*Escherichia coli* and *Staphylococcus* spp. are among the main bacterial contaminants of the eggshell surface of poultry eggs (Figure 2). In hatching eggs, Toghyani et al. [9] quantified *Escherichia coli* loads of up to 4.89 log_10_ CFU/g of eggshell. Shahein and Sedeek [10] reported *Staphylococcus* spp. counts of 3.77 log_10_ CFU/egg on the surface of freshly laid hatching eggshells, increasing to 4.17 log_10_ CFU/egg after four days of storage. Furthermore, the persistence of *Staphylococcus* spp. during incubation was demonstrated by Fouad et al. [11], who recorded counts of 3.59 log_10_ CFU/egg on day 7 and 4.04 log_10_ CFU/egg on day 14 of incubation in untreated eggs, evidencing the maintenance and increase in the bacterial load throughout embryonic development while simultaneously increasing the likelihood of bacterial penetration through the eggshell barriers and subsequent colonization of embryonic tissues.

*Escherichia coli* and *Staphylococcus* spp. were also detected in dead-in-shell embryos and day-old chicks discarded from commercial hatcheries due to deformities and omphalitis. According to Amer et al. [21], *Escherichia coli* was the most frequently isolated bacterium, accounting for 28 of the 78 bacterial isolates recovered, whereas *Staphylococcus* spp. accounted for 17 isolates. Other bacterial genera frequently detected on eggshells (Figure 2) were also identified, including *Salmonella* spp., *Proteus* spp., *Citrobacter* spp., and *Klebsiella* spp. Therefore, bacterial contamination of eggs may persist throughout incubation, facilitating the penetration of microorganisms through the eggshell and their dissemination into embryonic tissues. Consequently, omphalitis, embryonic mortality, and impaired sanitary quality of the produced chicks may occur. To mitigate these risks, sanitizing products should be applied to hatching eggs to reduce the bacterial load on the eggshell surface.

## 3. Antibacterial Control of Hatching Eggs

Bacterial control of hatching eggs is primarily achieved through sanitization, a technique designed to reduce the microbial load on the eggshell surface. Sanitization can be applied to a single egg or to thousands simultaneously, depending on the system configuration, and is typically carried out at the farm post-collection. However, some hatcheries are equipped with specialized systems for this purpose. The role of sanitization in hatching eggs is significant in operations ranging from small to large scale, and it becomes even more important under higher microbial challenges, as sanitization protocols can reduce the total eggshell bacterial load by more than 6 log_10_ units [7], helping to maintain embryonic viability and prevent mass contamination. When properly applied, egg sanitization is generally well tolerated and presents a favorable safety profile. However, its effects depend on the sanitizer’s characteristics, including its chemical composition, concentration, and interactions with the organic and inorganic components of the eggshell [22]. Sanitizers may induce varying degrees of alteration in the cuticle and the underlying eggshell structure [22,23]. These compounds may not compromise the structural integrity of the shell, may promote partial disruption of the cuticle, or, when more aggressive, may intensify damage to the cuticle, potentially affecting the protective function of the eggshell [22,23]. Table 1 summarizes the three main methods of egg sanitization currently in use.

**Figure 3:**
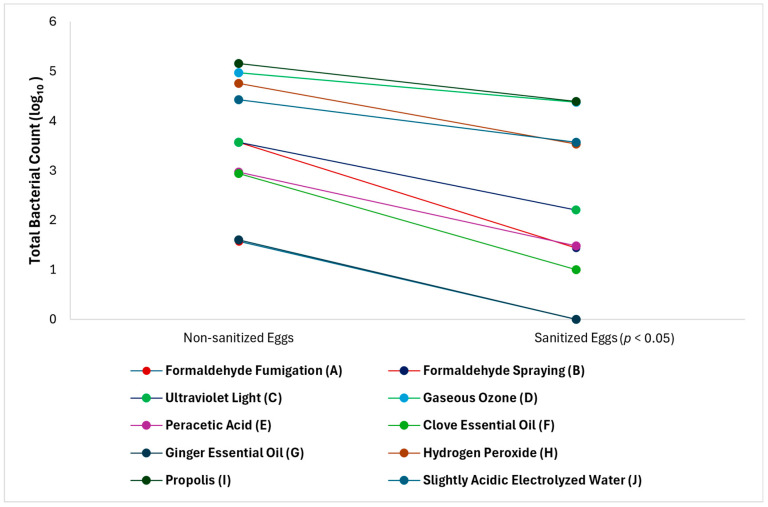
Significant reduction (*p* < 0.05) in total bacterial counts on eggs following treatment with different sanitizers, as reported in different studies. Source: Study A, Santos et al. [25]; Study B, Oliveira et al. [23]; Study C, Clímaco et al. [26]; Study D, Cilavdaroğlu et al. [27]; Study E, Melo et al. [28]; Study F, Oliveira et al. [29]; Study G, dos Santos [30]; Study H, Badran et al. [31]; Study I, Vilela et al. [32]; Study J, Liu et al. [33].

Bacterial control of hatching eggs continues to be performed predominantly through formaldehyde fumigation. Its popularity over several decades is attributed to its good antibacterial activity through alkylation of cellular macromolecules [24], combined with its ability to provide large-scale sanitization at a low cost. Nevertheless, other sanitizing agents are available for sanitizing hatching eggs. These include synthetic compounds, such as hydrogen peroxide and peracetic acid, as well as natural products, including propolis and plant-derived essential oils. As summarized in Figure 3, these alternatives, such as formaldehyde fumigation, can significantly reduce bacterial contamination on the eggshell surface, with varying levels of efficacy.

## 4. QACs and Their Antibacterial Activity

QACs constitute a class of organic compounds whose chemical structure is based on the NR_4_^+^ cation, in which the nitrogen atom is bonded to four organic substituents, such as alkyl, benzyl, or aryl groups [34], and are normally commercially available in liquid formulations for use as sanitizing agents. They have been evaluated for their sanitizing efficacy in applications targeting different livestock production sectors, including swine, cattle, and poultry [35,36,37]. QACs should be handled in compliance with established safety and preventive standards, including the use of personal protective equipment, even though exposure to these products represents a low risk to human health, as demonstrated by Osimitz and Droege [38]. Within the poultry sector, these compounds have been extensively evaluated for their sanitizing effectiveness in farms, poultry litter, hatching eggs, and hatcheries, primarily due to their antibacterial efficacy [37,39,40,41].

Reports showed that QACs exhibited both bacteriostatic and bactericidal activity against bacteria from distinct groups. This activity was demonstrated against *Escherichia coli*, *Salmonella* Enteritidis, *Salmonella* Typhimurium, and *Staphylococcus aureus*, with minimum inhibitory and bactericidal concentrations ranging from 0.00014% for *Salmonella* Enteritidis to 0.00937% for *Salmonella* Typhimurium [42]. QACs also produced inhibition zones against *Escherichia coli*, *Salmonella* Enteritidis, *Enterococcus faecalis*, *Klebsiella pneumoniae*, *Shigella* spp., and *Staphylococcus aureus* recovered from eggshell surfaces, with inhibition zones ranging from 10.33 mm for *Escherichia coli* to 20.00 mm for *Shigella* spp. [19]. These results demonstrate notable efficacy, mainly against enteric bacteria commonly associated with eggshell contamination. Other studies on poultry have also reported the efficacy of QACs against *Salmonella* spp., *Salmonella* Enteritidis, *Salmonella* Typhimurium, *Salmonella* Infantis, *Salmonella* Gallinarum, *Salmonella* Heidelberg, *Bacillus cereus*, *Staphylococcus aureus*, *Proteus mirabilis*, *Proteus vulgaris*, *Escherichia coli*, *Pseudomonas aeruginosa*, *Pasteurella multocida*, *Campylobacter jejuni*, and *Klebsiella oxytoca* [39,43,44,45,46,47,48,49].

Nadagouda et al. [50] reviewed the antibacterial mechanisms of action of QACs and reported that their antibacterial activity is associated with interactions between the cationic ammonium head group and negatively charged bacterial cell surfaces. This electrostatic interaction promotes adsorption of QACs onto the cell envelope, followed by the insertion of the hydrophobic alkyl chains into the lipid bilayer. As a result, membrane integrity is disrupted, thereby compromising bacterial viability and survival. In addition, Crnčević et al. [51] reported that the antibacterial action of QACs may also involve a combined effect on bacterial membranes and the inhibition of bacterial protein synthesis pathways. Therefore, QACs can act through more than one antibacterial mechanism to inactivate bacteria.

## 5. NaClO and Its Antibacterial Activity

NaClO (composed of sodium cations, Na^+^, and hypochlorite anions, ClO^−^) is one of the chlorine-based sanitizers [52] and is commercially available in liquid formulations, commonly referred to as bleach, for sanitizing. NaClO is considered safe for humans and is widely used and tested as a surface sanitizer, including in commercial poultry food processing plants and for sanitizing hatching eggshell surfaces, not only due to its low cost but also because of its antibacterial activity [6,53]. Its handling, like that of QACs, requires personal protective equipment.

NaClO can render the survival of different bacterial strains unfeasible. At concentrations ranging from 0.08 to 0.32% and with a contact time of 1 min, this compound exhibited a bactericidal effect against *Klebsiella pneumoniae*, *Klebsiella oxytoca*, *Acinetobacter baumannii*, *Acinetobacter pittii*, and *Pseudomonas aeruginosa* [54]. Abd-Elall et al. [55] reported that at a concentration of 5% and a contact time of 120 min, NaClO eliminated biofilms of *Salmonella* Enteritidis and *Escherichia coli*. At the same concentration, a reduced contact time of 60 min resulted in complete removal of the *Staphylococcus aureus* biofilm. The efficacy of NaClO against other *Salmonella* serovars, such as *Salmonella* Typhimurium, *Salmonella* Infantis, *Salmonella* Gallinarum, and *Salmonella* Braenderup, has also been reported [43,56].

NaClO can act against bacteria by inducing oxidative stress. According to Bridges et al. [57], exposure of bacteria to NaClO increased expression of genes involved in defense against reactive oxygen species, such as sodA, oxyR, and soxR, and activated genes associated with general stress responses, including *uspA*. In addition, significant alterations in the cellular redox state were observed, with a significant reduction in NADH and NADPH levels. Beyond its role in inducing oxidative stress, Wang et al. [58] demonstrated that this compound also exerts antibacterial activity at the bacterial cell membrane, increasing membrane permeability and promoting the leakage of ions and protons, ultimately leading to a loss of membrane potential.

## 6. Potential of QACs and NaClO to Reduce Bacterial Load on the Eggshell

### 6.1. Methodology for Article Search

The references used in this topic were identified through searches in the Google Scholar database in December 2025, to retrieve articles published between 2016 and 2025. The search was conducted in English and Portuguese, using 20 keyword combinations (10 in each language) with the advanced search option set to include all words. The keywords used to identify studies addressing the use of QACs were: quaternary ammonium compounds, quaternary ammonium, QAC, QACs, eggs, eggshells, hatching eggs, table eggs, sanitizers, disinfectants, sanitization, disinfection, washing, antibacterial agents, antimicrobial agents, and poultry. The keywords used to identify studies addressing the use of NaClO were: sodium hypochlorite, hypochlorite, chlorine-based sanitizers, chlorine compounds, NaClO, eggs, eggshells, hatching eggs, table eggs, sanitizers, disinfectants, sanitization, disinfection, washing, antibacterial agents, antimicrobial agents, and poultry. Articles retrieved from the first five pages of each search were reviewed. This page count was selected because, in subsequent pages, a substantial reduction was observed in the number of studies directly addressing this study’s objective. Furthermore, because multiple search strategies using different keyword combinations were employed, studies that appeared beyond the fifth page of one search could also appear among the first results of another, thereby reducing the likelihood of excluding relevant studies. The retrieved studies were screened based on their titles, abstracts, and full texts and included only when they met the eligibility criteria established for this review. The searches and data extraction were conducted independently by one of the authors (GdSO), who also established the initial datasets, using Microsoft Excel for data organization. These datasets were subsequently validated by the other three authors (LMdJ, CM, and VMdS).

The study focused on sanitizing chicken hatching eggs with QACs or NaClO, either alone or in combination with other agents. Original and conference papers published in English and Portuguese were included. This review primarily focused on sanitizing hatching eggs. However, studies involving table eggs were also considered to complement evidence on eggshell surface characteristics, particularly regarding bacterial control. As the evaluated compounds act directly on microorganisms present on the eggshell, regardless of the egg’s intended production purpose, these studies provide additional information on the sanitizers’ antibacterial efficacy, thereby contributing to a broader understanding of their potential applications. Nevertheless, conclusions regarding incubation performance, including embryonic development and hatchability, were based exclusively on studies involving hatching eggs. Additionally, studies addressing eggs from other poultry species, such as ostriches, turkeys, and ducks, were included. Review, books, book chapters, and any other materials that were not original research papers were excluded. Reports lacking sufficient details on the procedures, articles in languages other than English or Portuguese, studies whose full text was unavailable, studies unrelated to the research objective, and duplicate papers were also excluded. The searches were concluded once the conceptual scope of the topic had been adequately covered. Although the initial search could have retrieved almost 2000 papers, 984 articles addressing QACs were excluded based on the previously mentioned criteria, resulting in 16 studies selected for inclusion. Similarly, 987 articles focusing on NaClO were excluded, leaving 13 studies included in the final analysis. The included studies were organized in Mendeley (Elsevier) and subsequently exported to VOSviewer (version 1.6.20), where bibliometric analyses were conducted to provide a general characterization of the included literature.

As egg sanitization using QACs and NaClO remains underexplored, and because some of the available studies have been published in journals not indexed in major bibliographic databases, Google Scholar was selected as the search engine. Google Scholar is advantageous for research areas with a limited body of literature, as it simultaneously indexes peer-reviewed publications from multiple publishers and databases, enabling broader coverage of the available literature on egg sanitization with these compounds.

### 6.2. Main Findings: QACs

**Figure 4:**
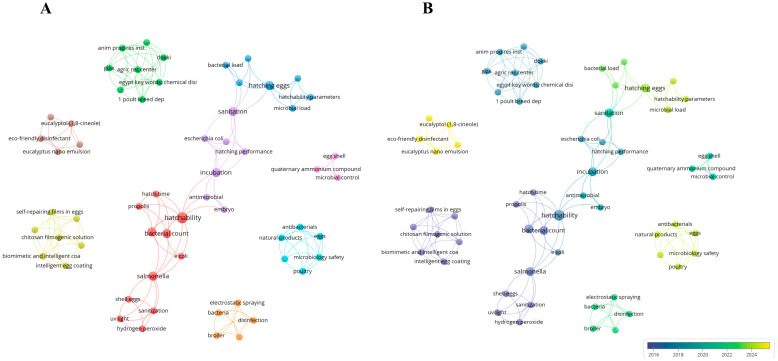
Keyword co-occurrence network of the included studies (**A**) and its temporal overlay (**B**) among the included studies on QACs. In Figure 4A, node size is proportional to keyword occurrence frequency, while links indicate co-occurrence relationships between keywords. Different colors represent thematic clusters. In Figure 4B, node colors indicate the average publication year associated with each keyword, ranging from earlier (blue) to more recent (yellow) studies.

The keyword co-occurrence network shows that hatchability has the largest node, indicating that it is the most recurrent term in the included literature (Figure 4A). In addition, the temporal overlay of the keyword co-occurrence network, as shown in Figure 4B, indicates that hatchability has been a central axis throughout the years (Figure 4B). The keyword hatchability was strongly associated with terms such as bacterial count, *Salmonella*, *Escherichia coli*, and sanitization, indicating that studies directly link egg bacteriological parameters to incubation performance. This finding is relevant because hatchability was not one of the evaluation parameters in the present review, nor was this term included in the adopted search strategies, thereby reinforcing its importance as a central target in this field of research. Despite this, the relationship between QACs and hatchability appears to be beneficial. Mustafa et al. [41] sanitized hatching eggs by spraying them with QAC at three concentrations (1:100, 1:200, and 1:300 mL/L in water). Eggs treated at the 1:300 concentration showed the highest mean hatchability values, whereas treatments at 1:100 and 1:200 resulted in numerically lower values. However, none of the treatments differed statistically from the non-sanitized control, indicating that the application of QAC, regardless of the concentration evaluated, did not impair the hatchability of hatching eggs.

**Figure 5:**
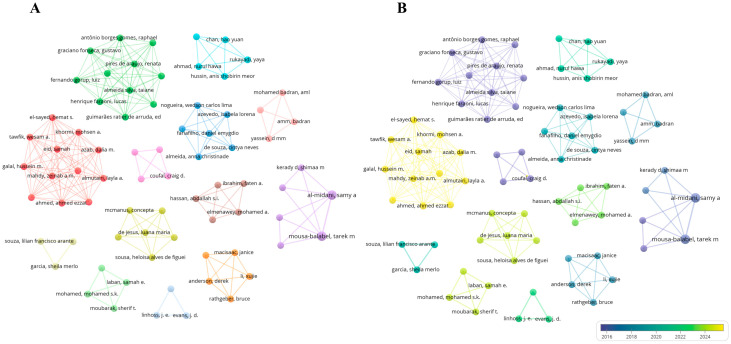
Co-authorship network showing collaboration among authors (**A**) and its temporal distribution (**B**) among the included studies on QACs. In Figure 5A, each node represents an author, node size is proportional to the number of publications, and links indicate co-authorship relationships. Different colors represent collaboration clusters among authors. In Figure 5B, node colors indicate the average publication year associated with each author, ranging from earlier (blue) to more recent (yellow) contributions.

The analysis of the co-authorship network revealed low interconnectivity among the different groups, indicating that authors maintain predominantly recurrent internal collaborations (Figure 5A). The temporal dimension evidenced the gradual inclusion of new authors (Figure 5B), especially in more recent years, suggesting the maintenance of research lines alongside the renewal and expansion of the groups.

**Figure 6:**
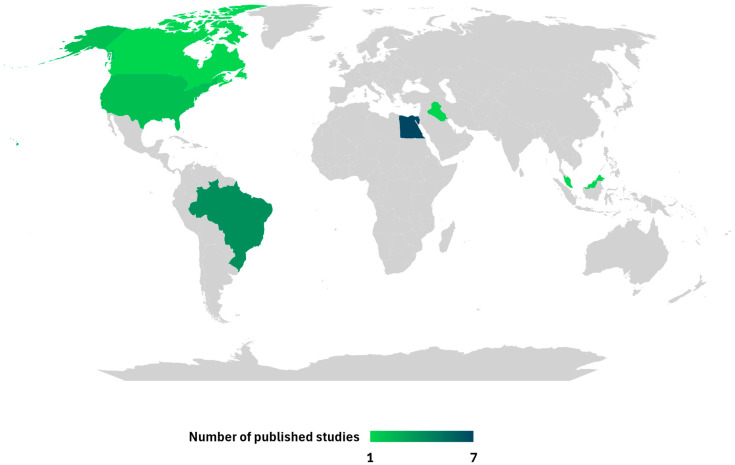
Geographic distribution of studies on egg sanitization using QACs. The countries highlighted on the map correspond to the locations where the studies included were conducted.

The studies originated mainly from Egypt, Brazil, and the United States (Figure 6) and were published in journals such as Poultry Science, Egyptian Poultry Science Journal, IJSBAR, and Microbiology Research. These findings highlight the important contributions of Egypt and Brazil to research on egg sanitization using QACs, as well as the central role of poultry-focused journals in disseminating this knowledge.

The evidence synthesized from a decade of independent studies indicates that nearly all trials reported a reduction in eggshell bacterial load at the concentrations and application methods evaluated (Table 2). The included studies comprised predominantly hatching eggs, although evidence from table eggs was also available. Positive antibacterial effects were observed in both categories, suggesting that the reduction in eggshell bacterial contamination is associated with the direct antimicrobial action of QAC-based sanitizers on the eggshell surface. Specifically, 93.75% of the studies reported a reduction in bacteria, whereas 6.25% did not observe a statistically significant difference. Reductions exceeding 4 log_10_ in *Salmonella* Enteritidis were reported by Al-Ajeeli et al. [59]. Mousa-Balabel et al. [60] recorded reductions of 88.00% in total bacterial counts and 86.05% in total coliform counts. Li et al. [61] described significant reductions in *Escherichia coli* counts. More recently, El-Sayed et al. [62] observed reductions in contamination by *Escherichia coli*, *Salmonella* Typhimurium, *Staphylococcus aureus*, and *Pseudomonas* spp.

The most frequently evaluated application methods were spraying and immersion, both of which yielded positive outcomes, with spraying emerging as the predominant technique across studies (Table 2). In addition, combined formulations such as QACs associated with chitosan or glutaraldehyde demonstrated enhanced efficacy, indicating additive or synergistic interactions that improve antibacterial performance. Silva et al. [42] reported that the association of chitosan with QACs resulted in a pronounced synergistic antibacterial effect, evidenced by a reduction of up to two logarithmic units in the minimum inhibitory concentration required to control *Salmonella* Enteritidis. Furthermore, chitosan–QAC coatings inhibited the adhesion and biofilm formation of *Salmonella* Enteritidis on eggshell surfaces at substantially lower QAC concentrations than those required when QACs were applied alone, supporting their role in improving eggshell decontamination efficiency.

The concentrations evaluated ranged from ppm levels to percentage solutions, with studies demonstrating antibacterial efficacy even at lower concentrations when appropriate application protocols were employed (Table 2). Valdo et al. [65], for example, evaluated the eggshell surface after applying QACs at 400 ppm and reported that total mesophilic bacteria, total coliforms, and *Pseudomonas* spp. were reduced to undetectable levels. Similarly, Li et al. [61] reported that applying a QAC–based sanitizer at 0.125% concentration significantly reduced the population of *Escherichia coli* penetrating the eggshell, suggesting that this sanitizer may effectively limit microbial penetration.

### 6.3. Main Findings: NaClO

**Figure 7:**
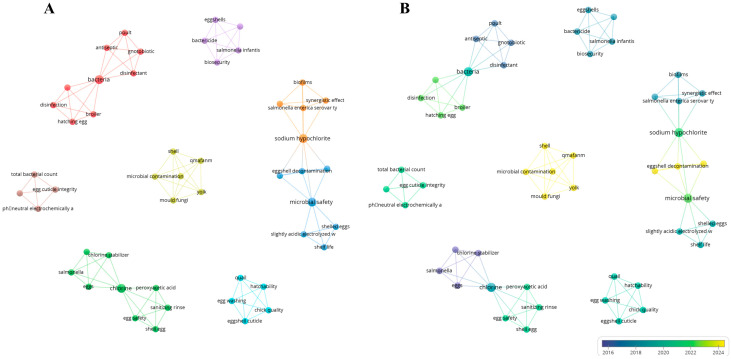
Keyword co-occurrence network of the included studies (**A**) and its temporal overlay (**B**) among the included studies on NaClO. In Figure 7A, node size is proportional to keyword occurrence frequency, while links indicate co-occurrence relationships between keywords. Different colors represent thematic clusters. In Figure 7B, node colors indicate the average publication year associated with each keyword, ranging from earlier (blue) to more recent (yellow) studies.

The keyword co-occurrence network reveals that scientific production is strongly focused on strategies for sanitization, microbiological control, and egg safety (Figure 7A). Over time, the analysis of the temporal distribution of keywords reveals a transition from a focus on isolated sanitization strategies to more integrated approaches that consider shelf life, synergistic effects, and impacts on final product quality (Figure 7B). In addition to the terms previously used in the paper search strategy, *Salmonella*, which was not included as a search term, emerged as one of the most frequent author keywords. This finding indicates that *Salmonella* spp. are among the main bacteria of interest in studies focused on the microbial control of table and hatching eggs, due to their high potential as sources of cross-contamination and the associated risks to both avian and human health. In this context, the results reported by Tenzin et al. [69] demonstrated that the application of NaClO at 200 mg/L of free available chlorine to the eggshell surface promoted a significant reduction in *Salmonella* Enteritidis, achieving complete elimination of detectable bacteria on the eggshell surface, with a reduction of 5.4 log_10_ CFU/egg compared with the untreated control.

**Figure 8:**
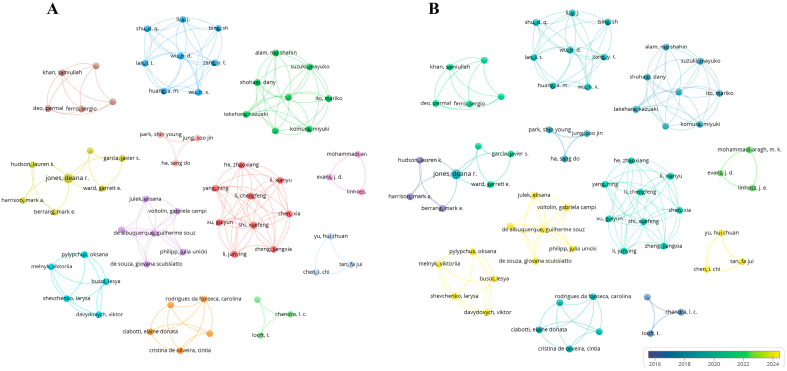
Co-authorship network showing collaboration among authors (**A**) and its temporal distribution (**B**) among the included studies on NaClO. In Figure 8A, each node represents an author, node size is proportional to the number of publications, and links indicate co-authorship relationships. Different colors represent collaboration clusters among authors. In Figure 8B, node colors indicate the average publication year associated with each author, ranging from earlier (blue) to more recent (yellow) contributions.

The co-authorship network highlights that scientific production occurs in independent cores, with strong internal interaction and limited articulation between groups (Figure 8A), and also shows that these cores undergo gradual renewal as the research line continues (Figure 8B).

**Figure 9:**
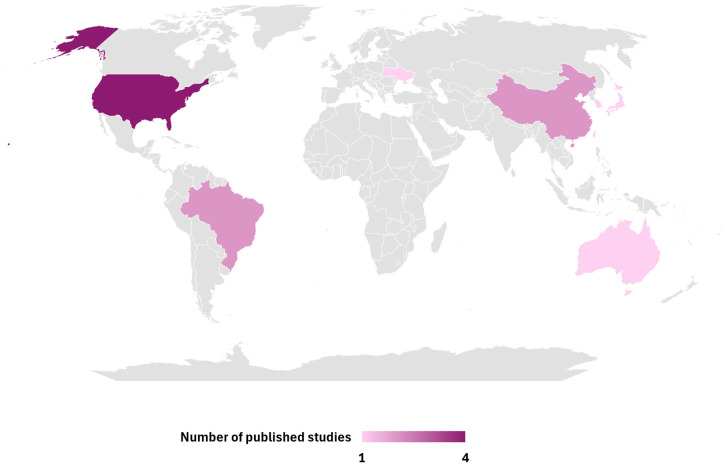
Geographic distribution of studies on egg sanitization using NaClO. The countries highlighted on the map correspond to the locations where the studies included were conducted.

The studies originated mainly from the United States, Brazil, and China (Figure 9) and were published in journals such as Poultry Science, Journal of Food Protection, and Avian Diseases. These findings highlight the contributions of the United States and Brazil to research on bacterial control in eggs using NaClO, as both countries have also contributed studies focused on egg sanitization with QACs. In addition, these findings emphasize the central role of not only poultry- but also food-focused journals in disseminating this knowledge.

Unlike studies on QAC-based compounds, studies investigating the use of NaClO have predominantly focused on table eggs rather than hatching eggs. The predominance of studies conducted with table eggs indicates that further investigations involving hatching eggs are still needed to strengthen the evidence base for incubation systems. Table 3 shows that NaClO was evaluated at different available chlorine concentrations. Despite this variation, the majority of studies (92.31%) reported a reduction in eggshell bacterial load, suggesting that NaClO can reduce eggshell bacterial contamination under different experimental conditions. Furthermore, positive antibacterial effects were reported in both table and hatching eggs, indicating that the efficacy of NaClO is consistent across different egg types. Reductions of 1.48 log_10_ CFU/mL for *Salmonella* Braenderup and 1.16 log_10_ CFU/mL for *Salmonella* Enteritidis on the eggshell surface were reported by Jones et al. [56]. Additionally, a reduction of 1.23 log_10_ CFU/mL in total bacterial counts on the eggshell surface was observed by Yu et al. [70].

Among the included studies, a similar distribution was observed regarding the application method, with some using immersion and others using spraying, and both approaches demonstrated effective application of the sanitizing agents (Table 3), which may reflect not only the efficiency of the sanitizer itself but also the ability of these methods to promote adequate contact between the sanitizer and the eggshell surface. Additionally, the use of NaClO in combination with other compounds does not appear to compromise its antibacterial efficacy. Davydovych et al. [77] washed and sanitized eggs using a sanitizer at a concentration of 0.5%, containing NaClO and sodium hydroxide, with an active chlorine content of 5.6% and observed that this sanitization effectively eliminated detectable colonies of mesophilic aerobic and facultative anaerobic bacteria from the eggshell surface immediately after treatment.

QAC- and NaClO-based sanitizers can reduce bacterial contamination on eggshells under the experimental conditions evaluated in the available studies. The repeated observation of positive antibacterial outcomes, together with the absence of recurrent sanitary failures, strengthens the evidence supporting the use of both sanitizers. Nevertheless, there remains a need for standardized and comparative trials to support the selection of practical application protocols.

Despite this review’s focus on bacterial control on the eggshell surface, the included studies suggest that sanitizers based on QACs and NaClO may induce alterations in the eggshell cuticle [70,76]. However, it remains unclear whether these modifications impair the functionality of this structure to an extent that results in relevant adverse effects on embryonic development and incubation performance. Part of this uncertainty stems from the fact that some studies have assessed the effects of these sanitizers indirectly, using egg weight loss as an indicator of cuticle integrity. In these studies, the use of QACs did not affect egg weight loss [61,67], whereas NaClO was associated with cuticle damage, although no detectable effects on this parameter were observed [76]. Furthermore, reports indicate that neither sanitizer caused significant reductions in hatchability [41,76], suggesting that either the absence of significant structural damage to the cuticle or alterations in this structure may not have been sufficient to impair this productive parameter under the conditions evaluated in each study [41,76]. This issue deserves special attention, as the eggshell structure is fundamental to embryonic development and survival. Therefore, studies investigating the sanitization of eggs with QACs and NaClO, while directly assessing eggshell integrity in association with incubation performance parameters, are needed to elucidate this relationship.

As observed in this review, QAC-based sanitizers have been more frequently evaluated in relation to incubation performance parameters, whereas studies involving NaClO have focused mainly on bacterial decontamination in table eggs. Therefore, the available literature remains insufficient to support well-supported comparisons between these sanitizers with respect to their effects on embryonic development, chick quality, hatchability, and post-hatch performance. This may also explain why incubation-related parameters were not included as primary outcomes in this review.

## 7. Conclusions

Sanitizers containing QACs or NaClO, either alone or in combination with other compounds, represent a useful option in addressing bacterial challenges associated with eggshell contamination, as the available studies indicate their effectiveness in reducing bacterial load on the eggshell. However, their performance depends on the sanitization protocol employed. These sanitizers are low-cost, effective, readily available, and easy to apply, making them especially attractive for poultry producers. Before using them, attention should be paid to handling procedures, and experimentally validated protocols or manufacturers’ recommendations must be strictly followed, since improper application may impair embryonic development. Research and review studies evaluating the effects of these compounds on poultry health and productive parameters are encouraged.

## Figures and Tables

**Table 1:** Main methods for the application of sanitizers to hatching eggs.

Method	Method Image	Method Description
Spraying	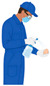	This method involves applying the product in liquid form with a manual sprayer, although a mechanized system may also be used.
Immersion	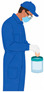	This method involves submerging the egg in a liquid solution in a vessel and then removing it.
Fumigation	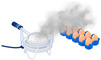	This method is generally carried out using a fumigator, in which the fumigant combusts, followed by volatilization and dispersion of the gas over the eggs.

**Table 2:** Effects of sanitizers containing quaternary ammonium compounds (QACs) on eggshell bacterial counts.

Study	Sanitizer	Egg Type	Concentration	Application	Bacterial Load
Al-Ajeeli et al. [59]	QAC	Table egg	200 ppm	Spraying	Reduced *
Mousa-Balabel et al. [63]	TH4	Hatching egg	0.5%	NI	Reduced *
Mousa-Balabel et al. [60]	TH4	Hatching egg	0.5%	NI	Reduced *
Li et al. [61]	QAC (Power Quat^®^)	Hatching egg	0.125%	Fumigation	Reduced *
Badran et al. [31]	TH4	Hatching egg	0.1–0.7%	Spraying	Reduced *
Nogueira et al. [64]	QAC	Hatching egg	0.1%	Immersion	NS
Valdo et al. [65]	QAC	Hatching egg	400 ppm	Spraying	Reduced *
Silva et al. [42]	QAC + chitosan coating	Table egg	QAC: 0.25–1.0%	Immersion	Reduced *
Chan et al. [4]	QAC	Table egg	100–200 ppm	Immersion	Reduced *
Mohammadi et al. [66]	BioShield 75 or Virocid	Hatching egg	0.50–0.75%	Spraying	Reduced *
Hassan et al. [19]	BioSentry 904	Hatching egg	NI	Spraying	Reduced *
de Jesus et al. [67]	QAC	Table egg	1%	Spraying	Reduced *
Maatouq et al. [68]	Aldekol GDA^®^	Hatching egg	0.5%	Spraying	Reduced *
Laban et al. [5]	Glutaraldehyde + QAC	Hatching egg	0.5%	Spraying	Reduced *
El-Sayed et al. [62]	TH4	Hatching egg	1:1000	Immersion	Reduced *
Mustafa [41]	QAC	Hatching egg	1:100–1:300	Spraying	Reduced *

Abbreviation: *, Significant effect; NI, not identified; NS, Not significant.

**Table 3:** Effects of sanitizers containing sodium hypochlorite (NaClO) on eggshell bacterial counts.

Study	Sanitizer	Egg Type	Sanitizer or Chlorine Concentration	Application	Bacterial Load
Hudson et al. [71]	NaClO	Table egg	170–200 ppm	Spraying	Reduced *
Sylte et al. [72]	NaClO	Hatching egg	0.85%	Immersion	Reduced *
Alam et al. [73]	NaClO	Table egg	150 ppm	Immersion	NS
Jung et al. [74]	NaClO	Table egg	50–300 ppm	Immersion	Reduced *
dos Santos Neto [75]	NaClO	Table egg	50–100 ppm	Immersion	Reduced *
Zang et al. [7]	NaClO	Table egg	10–26mg/L	Immersion	Reduced *
He et al. [76]	NaClO	Hatching egg	0.13%	Immersion	Reduced *
Tenzin et al. [69]	NaClO	Table egg	200 mg/L	Spraying	Reduced *
Jones et al. [56]	NaClO	Table egg	100–200 ppm	Spraying	Reduced *
Mohammadi et al. [66]	NaClO	Hatching egg	150 ppm	Spraying	Reduced *
de Souza et al. [6]	NaClO	Table egg	1%	Spraying	Reduced *
Davydovych et al. [77]	NaClO + NaOH	Table egg	0.5%	Washing	Reduced *
Yu et al. [70]	NaClO	Table egg	150 ppm	Spraying	Reduced *

Abbreviation: *, Significant effect; NS, not significant.

## Data Availability

No new data were created or analyzed in this study.
